# Beyond the genome: clinical challenges in diagnosing *LONP1*-related mitochondrial disorders

**DOI:** 10.3389/fcell.2026.1779332

**Published:** 2026-03-27

**Authors:** Qianni Jiang, Jing Duan, Chao Liu, Yuanyuan Zhu, Qi Zeng, Yuhui Hu, Zhiqiang Luo, Dezhi Cao, Jianxiang Liao, Li Chen

**Affiliations:** 1 Department of Neurology, Shenzhen Children’s Hospital, Shenzhen, China; 2 Shenzhen Pediatrics Institute of Shantou University Medical College, Shenzhen, China; 3 Shenzhen Maternity and Child Healthcare Hospital, Southern Medical University, Shenzhen, China; 4 Be Creative Lab Co., Ltd., Beijing, China

**Keywords:** adrenal crises, autosomal dominant, CODAS syndrome, LONP1, mitochondrial encephalopathy

## Abstract

**Background:**

*LONP1* encodes an ATP-dependent protease essential for maintaining mitochondrial homeostasis. *LONP1* variants have been associated with cerebral-ocular-dental-auricular-skeletal anomalies syndrome, pediatric cataract, congenital diaphragmatic hernia, and neurodevelopmental disorders; moreover, these variants can be inherited in both autosomal recessive and autosomal dominant modes.

**Methods:**

We conducted a retrospective analysis of the clinical data and genetic test results of a Chinese boy diagnosed as having mitochondrial encephalopathy. Subsequently, we evaluated the pathogenicity of candidate variants and conducted a literature review encompassing 47 cases of *LONP1* variants.

**Result:**

The proband was a 4.5-year-old boy who had experienced focal epilepsy seizures since birth. He presented with recurrent seizures and did not respond to anti-seizure medications. He showed global developmental delay, microcephaly, pachygyria, and hyperlactatemia. Initial genetic testing through single and trio whole-exome sequencing before 6 months of age yielded no conclusive results. Recurrent seizures and elevated lactic acid levels at 18 months of age prompted reanalysis with trio whole-exome sequencing, leading to the identification of a likely pathogenic variant in *LONP1*: c.901C>T (p.Arg301Trp). By 10 months of age, the patient had already developed primary adrenal insufficiency and experienced multiple adrenal crises triggered by respiratory infections, necessitating admission to the intensive care unit. The crises were effectively managed with hydrocortisone. However, despite intensive medical interventions, the patient succumbed to a metabolic crisis triggered by a severe respiratory infection at the age of 4.5 years.

**Conclusion:**

In this study, we discuss the clinical manifestations and genetic features of a pediatric patient with mitochondrial encephalopathy resulting from a rare *LONP1* variant, emphasizing the diagnostic and therapeutic challenges of mitochondrial disorders. Furthermore, our findings enhance the understanding of *LONP1*-related diseases and offer additional evidence supporting the autosomal dominant inheritance pattern of *LONP1*.

## 1 Introduction

*LONP1*, located on chromosome 19p13.3, encodes a mitochondrial matrix protein belonging to the Lon family of ATP-dependent proteases (Venkatesh et al., 2012). The protein participates in various mitochondrial biological processes, such as the degradation of misfolded and oxidatively damaged proteins, regulation of mitochondrial protein quality control, formation of the mitochondria–endoplasmic reticulum membrane contact site, and maintenance of cellular structural stability, and it is involved as a molecular chaperone in mitochondrial complex assembly as well (Venkatesh et al., 2012; Lu et al., 2007; Li et al., 2023).

*LONP1* variants are associated with various human disorders, including cerebral-ocular-dental-auricular-skeletal anomalies (CODAS) syndrome (OMIM: 600373) (Strauss et al., 2015; Dikoglu et al., 2015), pediatric cataract (Khan et al., 2015), neurodevelopmental disorders (NDD) (Young et al., 2025), and congenital diaphragmatic hernia (CDH) (Qiao et al., 2021). Notably, diseases resulting from *LONP1* variants show dual inheritance modes, with clinical studies reporting both autosomal recessive (AR) and autosomal dominant (AD) patterns.

CODAS syndrome is a rare multi-system developmental disorder inherited in an AR manner. Clinically, it is characterized by developmental delay, craniofacial dysmorphism, cataracts, ptosis, dental anomalies, hearing loss, short stature, and delayed skeletal maturation (Shebib et al., 1991). The association between *LONP1* and CODAS syndrome was first identified in 2015, with a total of 17 reported cases (Strauss et al., 2015; Dikoglu et al., 2015). Inui et al. reported an atypical case of CODAS syndrome linked to *LONP1* variants, presenting with developmental delay, cataracts, spasticity, regression, and cerebellar atrophy (Inui et al., 2017). This atypical case showed only two characteristic features of CODAS syndrome, but showed an overlap with Marinesco–Sjögren syndrome (Inui et al., 2017).

*LONP1* has been implicated in pediatric cataract, as reported in 2015 (Khan et al., 2015; Patel et al., 2017). In affected individuals, cataract is the predominant clinical manifestation prompting medical evaluation, with rare instances of concurrent organ developmental anomalies. In addition, prior studies have identified *LONP1* as a candidate risk gene for CDH (Qiao et al., 2021).

*LONP1* has been associated with neurodevelopmental disorders (NDD) characterized by developmental delay, epilepsy, dystonia, and cerebral atrophy, and some cases have exhibited typical features of mitochondrial disease (Nimmo et al., 2019; Peter et al., 2018). Unlike CODAS syndrome, *LONP1*-related NDD presents with mitochondrial encephalopathy features, without manifesting CODAS syndrome-specific skeletal and dental anomalies (Young et al., 2025). Cases of *LONP1*-related NDD can show both AD and AR inheritance patterns. In 2020, Besse et al. (2020) documented the first case of mitochondrial encephalopathy (ME) in the world, which was attributed to a *de novo* variant in *LONP1*. After their initial report, Young et al. (2025) identified five additional cases of NDD caused by AD inheritance of *LONP1*.

In this study, we present the clinical and genetic data of a Chinese boy with ME caused by the *LONP1* variant c.901C>T. To our knowledge, this is the first documented case of a *LONP1*-related disorder with an AD inheritance pattern in China. It provides further evidence for the AD inheritance pattern of *LONP1* and broadens the clinical phenotypic spectrum of *LONP1*-related disorders.

## 2 Materials and methods

### 2.1 Sample collection

This study was approved by the Ethics Committee of Shenzhen Children’s Hospital and performed strictly in accordance with the Declaration of Helsinki, with the welfare and rights of the patient prioritized throughout the research process. Informed consent was obtained from participants’ guardians. Clinical information of a 4.5-year-old Chinese boy diagnosed with ME was collected, and genomic DNA was extracted from peripheral blood samples of the proband and his parents in accordance with standard protocols.

### 2.2 Trio whole-exome sequencing

First, 3 mL of peripheral blood was collected into EDTA tubes from the proband and his parents, and then, 50 ng genomic DNA from each sample was fragmented to an average size of 200 bp using NEB Next dsDNA Fragmentase (New England Biolabs, Ipswich, MA, United States). After end repair and A-tailing, the DNA fragments were amplified by PCR and then subjected to hybridization capture by SureSelect Human All Exon V6 (Agilent, San Diego, United States), according to the manufacturer’s protocol. The libraries were quantified by qPCR and subsequently sequenced on the Novaseq 6000 platform (Illumina, San Diego, United States) with 150-bp pair-end sequencing. The sequencing reads were aligned with the human reference genome (UCSC hg19) using the Burrows–Wheeler Aligner, followed by variant calling with GATK (https://software.broadinstitute.org/gatk/). Variant annotation and interpretation were performed using the Ensembl Variant Effect Predictor. The analysis incorporated data from the following resources: OMIM (http://www.omim.org), ClinVar (http://www.ncbi.nlm.nih.gov/clinvar), 1000 Genomes Project (https://www.internationalgenome.org), gnomAD (http://gnomad.broadinstitute.org/), and dbSNP (http://www.ncbi.nlm.nih.gov/snp). *In silico* prediction algorithms included REVEL, SIFT (http://sift.jcvi.org), MutationAssessor (http://mutationassessor.org), and CADD (http://cadd.gs.washington.edu). The pathogenicity of the candidate variants was assessed according to the guidelines of the American College of Medical Genetics (ACMG) for the interpretation of sequence variants.

### 2.3 Sanger sequencing

The candidate variants were validated by Sanger sequencing. PCR was conducted with Premix Taq™ Hot Start Taq® (Takara, Osaka, Japan) under the following conditions: initial denaturation at 95 °C for 5 min, followed by 34 cycles of denaturation at 95 °C for 30 s, annealing at 58 °C for 30 s, and extension at 72 °C for 30 s, with a final extension at 72 °C for 10 min. PCR products were purified and sequenced using an ABI 3730XL DNA Analyzer with the BigDye™ Terminator Cycle Sequencing Kit (Applied Biosystems, Foster, CA, United States). Using NM_004793.4 as the reference sequence, we analyzed the Sanger sequencing results using Chromas software (Version 2.6.6, Technelysium Pty Ltd., Australia).

### 2.4 Protein model

The three-dimensional (3D) structure of the LONP1 protein was generated by SWISS-MODEL (https://swissmodel.expasy.org/interactive) and visualized by PyMOL (The PyMOL Molecular Graphics System, Version 2.0 Schrödinger, LLC), based on the generated structure of *LONP1* (PDB ID: 7oxo.1), A as the template (Figure 4A).

## 3 Results

### 3.1 Clinical course

The proband was a 4.5-year-old boy, the first live-born child of healthy, non-consanguineous parents. He was delivered by cesarean section at 37 weeks’ gestation, and the perinatal course was uncomplicated. His birth weight was 2,350 g, which is below the 2500 g threshold for normal birth weight; thus, he was identified as a low-birth-weight infant. The first pregnancy of the parents ended in a spontaneous abortion at 8 weeks of gestation.

**FIGURE 1:**
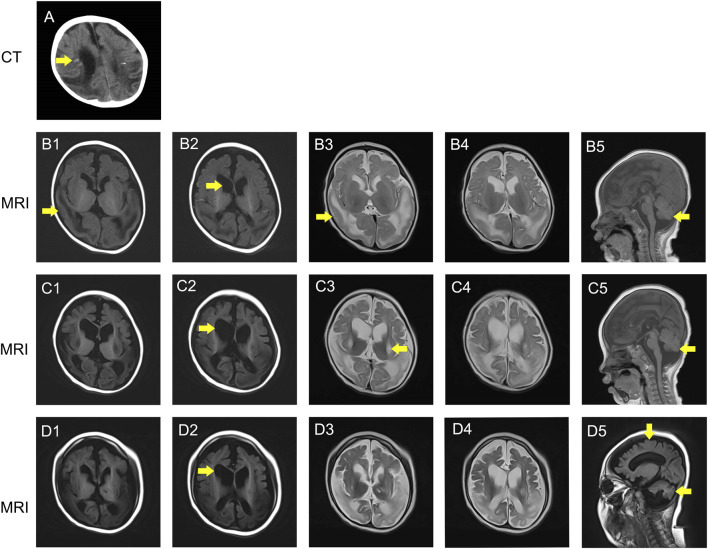
Neuroimaging results of the patient. **(A)** CT result of the brain (7 months of age): Small irregular calcifications next to the lateral ventricle. **(B1-D5)** MRI result of the brain. (**(B1-B5)** 3 months old, **(C1-C5)** 1.25 years old, **(D1-D5)** 4 years old): The MRI showed pachygyria in the temporal lobes **(B1, B3)**. Brain MRI performed at 3 months, 1 year, and 3 months, and 4 years of age revealed progressive whole brain atrophy **(B5, C5, and D5)** respectively).

Within hours after birth, the infant developed focal seizures, which remained the predominant seizure type throughout his first year. Phenobarbital and levetiracetam were sequentially introduced but failed to achieve seizure control. Whole-exome sequencing was performed at 6 days of age; however, no variants that could explain the patient’s phenotype were identified. A cranial MRI performed at 10 days of age showed extensive bilateral pachygyria (Figures 1 B1,B3).

At 3 months of age, EEG revealed hypsarrhythmia predominant over bilateral posterior regions with frequent focal seizures. Sequential trials of topiramate, lamotrigine, and vigabatrin, followed by a ketogenic diet introduced at 4 months of age, failed to reduce seizure frequency; the child continued to experience >20 focal seizures daily. MRI showed diffuse pachygyria accompanied by bilateral ventricle enlargement. No phenotype-explaining variants were identified by trio whole-exome sequencing performed at 4 months of age.

Between 7 and 9 months of age, a 6-week oral prednisone taper markedly reduced seizure frequency. After corticosteroid withdrawal at 10 months, patchy hyperpigmentation appeared over both knees and the scrotum, prompting endocrine evaluation. Remarkably, basal serum cortisol was 0.80 μg/dL (reference: 1.70–10.80 μg/dL) and plasma adrenocorticotropic hormone (ACTH) was 1,547 pg/mL (reference: 7.20–63.30 pg/mL), suggesting a tentative diagnosis of primary adrenal insufficiency. Meanwhile, the levels of dihydrotestosterone, dehydroepiandrosterone, 17-hydroxyprogesterone, aldosterone, and testosterone were normal, and findings of MRI were unremarkable. Oral hydrocortisone (8 mg/m^2^/day) was initiated, normalizing cortisol and ACTH within 6 weeks. Valproic acid, introduced at 10 months of age, markedly exacerbated seizures and induced hepatotoxicity; both resolved promptly after drug withdrawal.

At 15 months of age, the child developed new seizure types—myoclonic jerks and tonic spasms—accompanied by persistently elevated serum lactate levels and progressive proximal muscle wasting, raising clinical suspicion of ME. Although skeletal muscle biopsy was non-diagnostic (Supplementary Figure S1) and quantitative plasma amino acid and urinary organic acid profiles were unremarkable, empirical treatment with a mitochondrial cofactor regimen (co-enzyme Q10 100 mg once daily, L-carnitine 50 mg/kg once daily, thiamine 300 mg once daily, riboflavin 100 mg once daily, and taurine 1,000 mg twice daily), together with clonazepam and piracetam, was initiated. Over the subsequent 4 weeks, seizure frequency decreased by ∼40%, suggesting a partial therapeutic response without adverse effects, the mitochondrial cofactor regimen was continued until the child reached 4.5 years of age. MRI at this time showed pachygyria and significant cerebral and cerebellar atrophy (Figures 1 C1–C5). Based on the diagnosis of ME, we reanalyzed data of trio whole-exome sequencing performed at 4 months of age. Fortunately, this time we identified the *LONP1* variant: c.901C>T (p.Arg301Trp), thus resulting in the diagnosis of ME.

**FIGURE 2:**
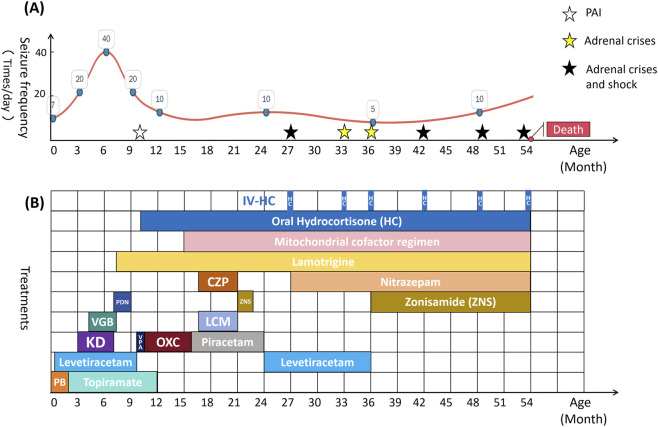
The patient’s clinical courses of major symptoms, treatments, and outcomes. **(A)** The frequency of epileptic seizures and progression nodes of the disease in the patient. The white star in the graph indicates primary adrenal insufficiency (PAI), the yellow stars indicate adrenal crisis, and the black stars indicate necessitated ICU admissions for shock. **(B)** The patient’s medication treatment course. PB, phenobarbital; VGB, vigabatrin; PDN, prednisone; VPA, sodium valproate; OXC, oxcarbazepine; LCM, lacosamide; CZP, clonazepam; NZP, nitrazepam; HC, hydrocortisone [the dose of intravenous drip HC (IV-HC) up to 100 mg/m^2^/day].

Since 2 years of age, five adrenal crises triggered by respiratory infections necessitated multiple ICU admissions for shock and metabolic decompensation. He required invasive mechanical ventilation two times. Vasopressor support (adrenaline and noradrenaline) helped maintain blood pressure, and midazolam helped control status epilepticus. High-dose hydrocortisone (up to 100 mg/m^2^/day) effectively reversed each crisis. Cerebral atrophy worsened on MRI at the age of 4 (Figure 1 D5). Regrettably, as a result of a severe metabolic crisis triggered by a pulmonary infection, the patient’s parents chose to discontinue treatment, resulting in his death at 4.5 years of age (Figure 2).

The patient never reached typical developmental milestones. He showed no auditory or visual tracking when presented with sound and moving objects, and his brainstem auditory evoked potentials at 2 months of age indicated bilateral auditory pathway dysfunction. Although he briefly showed some head-lifting ability at 2 months of age, he regressed and lost this ability by 4 months of age. His head circumference has consistently and always remained below −2 standard deviations. Persistent nonverbalism was observed in the patient throughout his life, accompanied by complete inability to sit independently, stand unassisted, or execute fundamental self-care tasks. None of these critical functional milestones was attained before his demise at 4.5 years of age.

### 3.2 A *de novo* missense variant of *LONP1*

**FIGURE 3:**
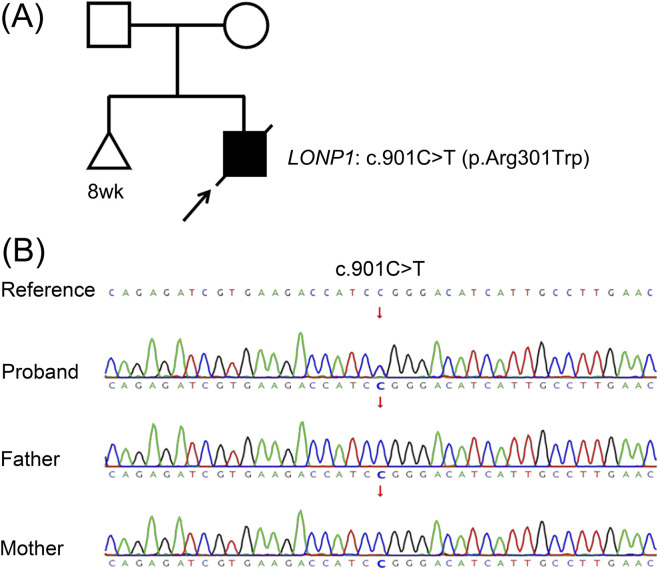
**(A)** The pedigree of the family: The outcome of the first pregnancy was spontaneous abortion. Born from the second pregnancy, the proband was the first live-born child of the parents and presented with recurrent seizures, microcephaly, pachygyria, developmental delay, hyperlactatemia, and hypoadrenocorticism. **(B)** Sanger sequence chromatogram of *LONP1*. Sanger sequencing showed that c.901C>T (p.Arg301Trp) was heterozygous in the proband, and the variant was not detected in the parents.

Trio whole-exome sequencing revealed a heterozygous variant of *LONP1* in the proband: NM_004793.3 c.901C>T (p.Arg301Trp). The REVEL score was 0.563. Identity by descent (IBD) analysis based on Trio WES data confirmed the paternity and maternity, and Sanger sequencing confirmed that his parents had the unmutated, wild-type variant of the gene (Figure 3; PS2). The variant was not found in population databases, such as gnomAD, EXAC, and 1000 Genomes Project (PM2_Supporting). However, it has been reported in the Human Gene Mutation Database. Besse et al. described a *de novo* variant, *LONP1* c.901C>T (p.Arg301Trp), in a pediatric patient diagnosed with ME (PS4_supporting). Functional experiments revealed increased hydrolytic activity of the mutated *LONP1*, indicating that this variant has gain-of-function mutation (Besse et al., 2020). In addition, a *de novo LONP1* variant (c.902G>A, p. Arg301Gln) was reported by Young et al. (2025) in a 27-year-old woman with NDD. However, as this variant was classified as a VUS, PM5 was not applicable in the current assessment. The ACMG guidelines identify this variant as likely pathogenic (PS2+PM2_supporting + PS4_supporting).

**FIGURE 4:**
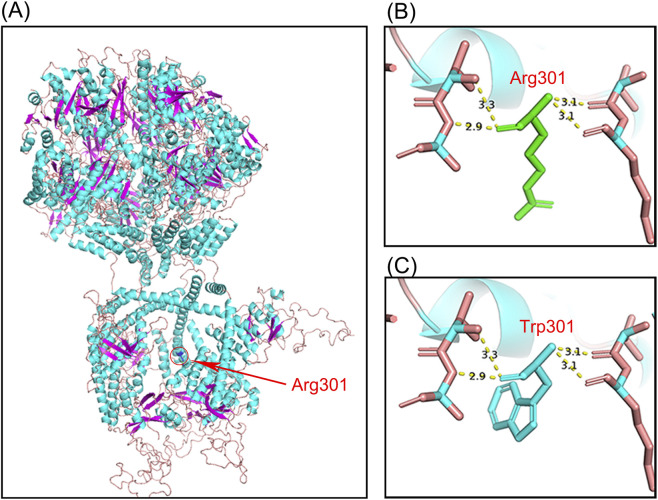
The protein structure of LONP1. **(A)** The red arrow points to the position of Arg301 in the LONP1 protein model. **(B)** Stick models show the amino acids around Arg301; four hydrogen bonds are formed among the four residues (Val297, Lys298, Ile304, and Ala305) surrounding Arg301. **(C)** Stick models show the amino acids around Trp301; four hydrogen bonds are formed among the four residues (Val297, Lys298, Ile304, and Ala305) surrounding Trp301.

Researchers used SWISS-MODEL to develop the three-dimensional (3D) protein structure of LONP1 (Figure 4). The predicted 3D structure of the mutant protein was not significantly different from that of the wild-type protein.

### 3.3 *LONP1*-associated diseases exhibit clinical and genetic heterogeneity

**FIGURE 5:**
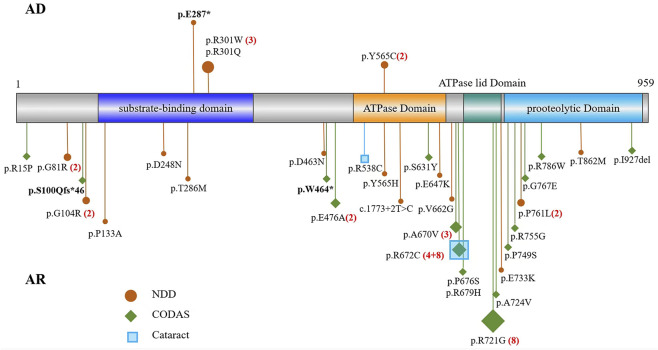
Schematic of *LONP1* and the localization of the variants of *LONP1* identified in previous reports and in this study. Variants above the axis exhibit an AD inheritance pattern, whereas those below the axis exhibit an AR inheritance pattern. The red circles correspond to NDD cases, the green diamonds to CODAS syndrome cases, and the blue squares to cataract cases. The numerical values following the * represent the reported cases with the same variants.

A comprehensive literature search was conducted using PubMed, Web of Science, and some Chinese databases, such as CNKI and WanFang up to October 2025. The search incorporated combinations of the following key terms: “CODAS syndrome” and “*LONP1* gene.” Considering the prior research designating *LONP1* as a candidate risk gene for CDH (Qiao et al., 2021), cases of CDH were not included in this study. We summarized 47 documented cases of *LONP1*-related diseases (Supplementary Table S1), among which 7 were inherited in an AD manner and 40 in an AR manner. We have summarized the variants of these 47 cases (Figure 5); there were 29 missense variants, 2 nonsense variants, 1 deletion, and 1 frameshift variant.

Clinical data were available for 37/47 cases of *LONP1*-related diseases. There were 16/37 cases of CODAS syndrome, 10/37 cases of *LONP1*-related NDD with an AR inheritance pattern (NDD-AR), 7/37 cases of *LONP1*-related NDD with an AD inheritance pattern (NDD-AD), and 4/37 cases of pediatric cataract. Among the 16 cases of CODAS syndrome (Strauss et al., 2015; Dikoglu et al., 2015; Khan et al., 2015; Shebib et al., 1991; Inui et al., 2017; Patel et al., 2017; Tang et al., 2023; Khan and AlBakri, 2018), the major clinical manifestations were cataracts (15/16), skeletal anomalies (15/16), craniofacial anomalies (14/16), developmental delay (13/16), auricular deformities (11/16), ptosis (5/16), and dental anomalies (5/16). Among the 4 cases diagnosed with pediatric cataract (Khan et al., 2015; Patel et al., 2017), the key clinical features comprised cataracts (4/4), craniofacial anomalies (3/4), ptosis (3/4), auricular deformities (3/4), developmental delay (2/4), and skeletal anomalies (1/4). This observation shows a significant overlap in clinical manifestations among patients with CODAS syndrome and pediatric cataract cases.

**FIGURE 6:**
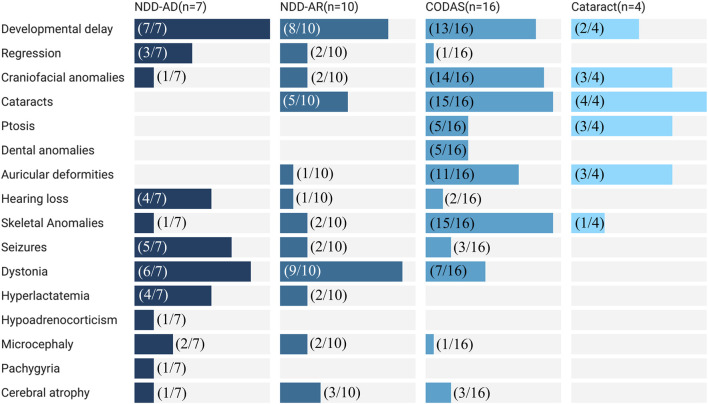
Clinical feature distribution of LONP1-related diseases across different subgroups.

Among *LONP1*-related NDD cases (Young et al., 2025; Nimmo et al., 2019; Peter et al., 2018; Besse et al., 2020; Hannah-Shmouni et al., 2019) encompassing both AD and AR inheritance patterns, the most prevalent clinical manifestations were developmental delay (7/7 cases in NDD-AD, 8/10 cases in NDD-AR), seizures (5/7 cases in NDD-AD, 2/10 cases in NDD-AR), and dystonia (6/7 cases in NDD-AD, 9/10 cases in NDD-AR). Clinical manifestations of NDD-AD and NDD-AR cases show significant similarity. Notably, ptosis and dental anomalies, two typical phenotypic features of CODAS syndrome, were absent in all *LONP1*-related NDD cases (Figure 6).

## 4 Discussion and conclusions

In this study, we present a case of a 4.5-year-old Chinese boy who experienced recurrent seizures, microcephaly, pachygyria, developmental delay, and hyperlactatemia. Trio-exome sequencing identified a *de novo* variant in *LONP1*, c.901C>T (p.Arg301Trp). The ACMG Standards and Guidelines (Richards et al., 2015) identify this variant as likely pathogenic, which may explain the phenotype of the patient. Reports of *LONP1*-related NDD with an AD inheritance pattern are scarce. We reviewed previous studies on *LONP1*-related diseases and identified six cases of *LONP1*-related NDD with an AD inheritance pattern. Consequently, our case is the first instance of *LONP1*-related NDD with AD inheritance reported in a Chinese individual and the seventh reported case globally. Notably, all reported variants associated with the seven cases of *LONP1*-related NDD with AD inheritance were *de novo* heterozygous variants, including c.859G>T, c.902G>A, c.1694A>G, c.1693T>A, and three instances of c.901C>T (Young et al., 2025; Besse et al., 2020).

The c.901C>T variant is the most commonly observed variant in cases of *LONP1*-related NDD with an AD inheritance pattern. The three patients carrying the c.901C>T variant showed similar clinical features, including recurrent seizures after birth, hyperlactatemia, and severe encephalopathy symptoms. MRI showed pachygyria in two of these patients. For one of these patients, the prognosis was dismal, with fatality reported due to respiratory infection at 12 months of age (Besse et al., 2020). The other patient, whose case was reported in 2025, remains alive; however, the follow-up duration is currently short, with the latest follow-up data being 2 months old (Young et al., 2025). The mitochondrial cofactor regimen and high-dose hydrocortisone administered during adrenal crises enabled the patient to survive to 4.5 years of age.

A noteworthy observation is that our patient presented with primary adrenal insufficiency (Bornstein et al., 2016) and received long-term hydrocortisone treatment. He was admitted to the ICU multiple times for adrenal crises triggered by infections, necessitating high-dose hydrocortisone therapy. PAI is a group of disorders characterized by impaired production of cortisol and other steroid hormones by the adrenal cortex (Güran, 2017). Glucocorticoid and mineralocorticoid replacement therapy is the mainstay of PAI management. According to clinical guidelines, all patients diagnosed with PAI should receive glucocorticoid therapy, whereas those with concomitant aldosterone deficiency require mineralocorticoid supplementation (Lewis et al., 2024). Mineralocorticoids play a key role in regulation of blood pressure and electrolyte balance. Compared to patients with secondary adrenal insufficiency, those with PAI have a higher risk of adrenal crisis (D et al., 2019). Our patient experienced recurrent adrenal crises, and thus, we deemed mineralocorticoid supplementation potentially beneficial.

To our knowledge, PAI has not been previously reported in *LONP1*-related cases. Notably, adrenal insufficiency has been reported in mitochondrial disorders, particularly in Pearson syndrome and Kearns–Sayre syndrome (Corkery-Hayward and Metherell, 2023), characterized by hyponatremia, hyperkalemia, hypoglycemia, skin hyperpigmentation, and elevated plasma ACTH levels (Siri et al., 2023). Human protein atlas information (The Human Protein Atlas) shows that *LONP1* mRNA is expressed at high levels in the adrenal gland. Our findings suggest that PAI is correlated with the expression of the *LONP1* variant. Moreover, the adrenal gland is an endocrine organ with high energy demands, and infection and stress have the potential to trigger adrenal crises (Bornstein et al., 2016). Our patient experienced recurrent adrenal crises triggered by infections, which were effectively managed with timely hydrocortisone therapy. The patient reported by Besse et al. succumbed to a respiratory infection at the age of 12 months (Besse et al., 2020). It is unclear whether the cause of death was associated with an infection-triggered adrenal crisis. Despite experiencing multiple episodes of metabolic decompensation and adrenal crises that required ICU admissions since 2 years of age, our patient survived until 4.5 years of age, achieving longer survival than noted in previous cases. This may be attributed to the early identification of PAI and prompt intervention for adrenal crises. The treatment experience of our patient can offer valuable insights for future patients with similar conditions.

*LONP1* has been associated with a spectrum of disorders, including CODAS syndrome, NDD, CDH, and pediatric cataracts. A review of 37 cases associated with *LONP1* revealed that CODAS syndrome and *LONP1*-related cataract are inherited in an AR manner and show significant clinical overlap, including developmental delay, craniofacial anomalies, cataracts, ptosis, auricular deformities, and skeletal anomalies (Strauss et al., 2015; Dikoglu et al., 2015; Khan et al., 2015; Shebib et al., 1991; Inui et al., 2017; Patel et al., 2017; Tang et al., 2023; Khan and AlBakri, 2018). The association between *LONP1* and CODAS syndrome was initially identified in 2015 (Strauss et al., 2015; Dikoglu et al., 2015). Considering the high similarity in clinical phenotypes between CODAS syndrome and pediatric cataract, we believe that cases diagnosed with pediatric cataract in the 2015 and 2017 studies may actually be CODAS syndrome cases. *LONP1*-related NDD primarily manifests as developmental delay, seizures, dystonia, hyperlactatemia, and in some cases, is accompanied by developmental regression and cerebral atrophy (Young et al., 2025; Nimmo et al., 2019; Peter et al., 2018; Besse et al., 2020; Hannah-Shmouni et al., 2019). However, typical features of CODAS syndrome, such as dental anomalies, ptosis, auricular deformities, and craniofacial anomalies, are less common. Variations in the phenotypic spectrum between *LONP1*-related NDD and CODAS syndrome indicate that *LONP1*-related NDD is a distinct disorder from CODAS syndrome. It is notable that *LONP1*-related NDD exhibits both AD and AR inheritance patterns (Supplementary Table S1). Despite the different inheritance patterns, the phenotypes of these cases show significant similarity.

Although the molecular mechanisms underlying *LONP1*-related diseases are not fully elucidated, it is certain that this gene is essential for sustaining life. Inactivation of LONP1 in mice embryonic cardiac tissue and/or lung epithelium resulted in embryonic or neonatal fatality (Qiao et al., 2021; Zhao et al., 2022). LONP1 functions as both a molecular chaperone and a protease and is instrumental in regulating multiple cellular pathways in response to environmental cues (Li et al., 2023; Li et al., 2017). In CODAS syndrome, functional experiments on certain *LONP1* variants have shown reduced enzyme activity compared with the wild type, with S3 cleavage ranging from 19% to 39% of wild-type activity, indicating a loss of function (Strauss et al., 2015). In AR inheritance cases of *LONP1*-related NDD, variants like (p.Tyr565His) and (p.Pro761Leu) have shown decreased proteolytic activity, with (p.Tyr565His) also showing impaired binding to the TFAM substrate, indicating a loss of function (Nimmo et al., 2019; Peter et al., 2018). Although the variants in both CODAS syndrome and *LONP1*-related NDD with an AR inheritance pattern are primarily loss-of-function variants, their variants show differences in their spatial distribution. In the 3D protein tertiary structure, CODAS syndrome is concentrated in a cluster at the periphery of the proteolytic core, whereas *LONP1*-related NDD variants are more diffusely distributed (Young et al., 2025).

In contrast, the molecular mechanisms of *LONP1*-related NDD with an AD inheritance pattern indicate a gain of function. In *LONP1*-related NDD with AD inheritance, functional studies of the p. Arg301Trp variant revealed increased *LONP1* proteolytic activity accompanied by a concomitant loss of chaperone function. This was associated with significant reductions in LONP1 proteolytic targets (TFAM, PINK1, and phospho-PDH E1α), as well as decreased levels of the mitochondrial ribosome subunits MRPL44 and MRPL11. These changes were accompanied by reduced activity and protein levels of oxidative phosphorylation complex I and IV subunits (Besse et al., 2020; Thomas et al., 2014). Consequently, the experimental findings of the p. Arg301Trp variant suggest a gain of function (Besse et al., 2020). Overall, the molecular mechanisms of LONP1 are intricate, and current evidence is insufficient. Further functional investigations are required to elucidate its mechanisms.

In conclusion, we identified a *LONP1* variant, c.901C>T, in a patient with ME. This finding expands the spectrum of diseases linked to *LONP1* variants and confirms the pathogenicity of dominant variants in *LONP1*. In addition, we emphasize that monitoring adrenal cortical function in affected patients and promptly managing adrenal crises are critically important for improving their survival outcomes and prognosis.

## Data Availability

The original contributions presented in the study are included in the article/Supplementary Material, further inquiries can be directed to the corresponding author.
