# Supplementary material for: Beyond the genome: clinical challenges in diagnosing LONP1-related mitochondrial disorders
Source: Front Cell Dev Biol. 2026 Mar 27;14:1779332. doi: 10.3389/fcell.2026.1779332 (PMC13066256; doi:10.3389/fcell.2026.1779332)
Supplement: Supplementary file 2 [file DataSheet1.docx]

**Supplementary Figure**


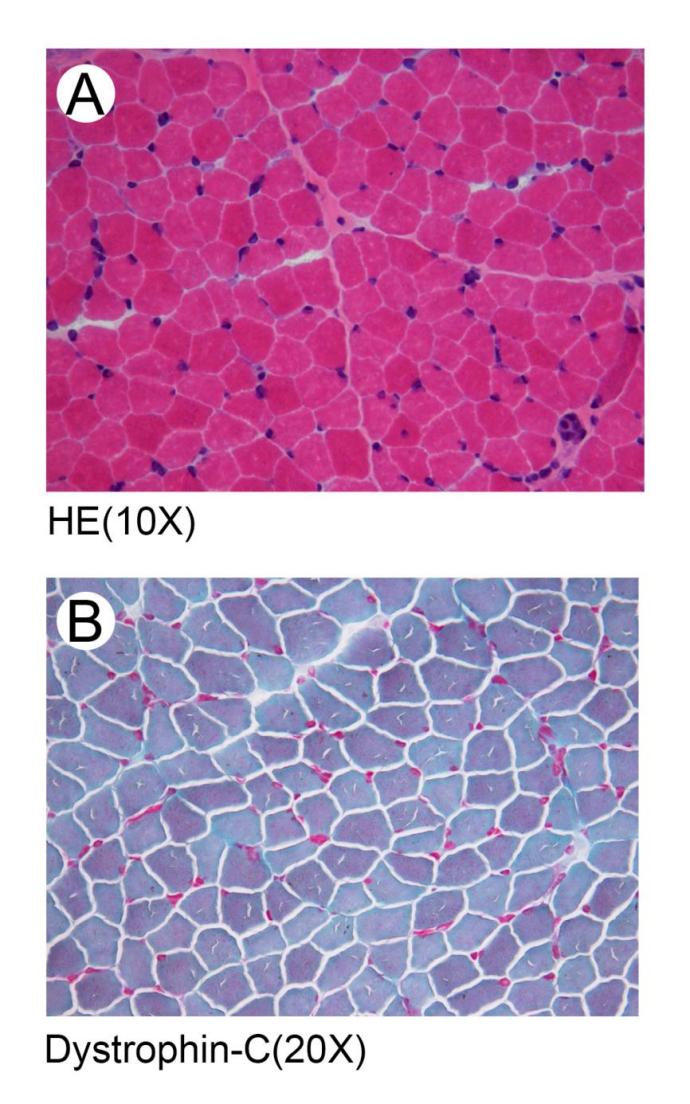


**Supplementary Figure 1.** Pathological biopsy (right thigh muscle): Mild muscle damage was observed.

**(A)** HE 10X: revealed mild hyperplasia of perimysium fibroadipose tissue. The size of muscle fibers showed slight inconsistency, with a few fibers exhibiting degeneration. No regenerated muscle fibers were detected. No ragged red fibers were detected.

**(B)** Dystrophin-C 20X: No ragged red fibers were detected.
